# The Code of Ethics

**Published:** 1867-06

**Authors:** 


### THE CODE OF ETHICS.

THE Code of ethics of the "American Dental Association" has, as far as known, met with a favorable reception by the local societies which have taken it into serious consideration. The Ohio State Society adopted it, though its own, previously adopted, covered the same grounds. The "Mississippi Valley Association" had her mind made up, and adopted it by a unanimous vote, without debate. Several of the eastern societies, and nearly, if not all in the west and south have recognized it. It has met with a little opposition in certain quarters, as was expected by its friends and advocates. It met with some in the American Association at the Boston meeting, or, as another has remarked, it "elicited much discussion, and no little opposition." The amount of discussion was really small, in comparison with that of other matters that came before the meeting, and in view of the great importance of the subject; and the "opposition" showed but a small force at the final vote.

A few general thoughts on this subject may not be out of place.

That something of the kind was needed, had been indicated for years. Prominent members of the profession, at various times and places, had written ably and earnestly on the subject of professional ethics. Societies and associations had recognized the principle that their members were responsible, *as members*, for their professional conduct, and liable to censure for violation of professional ethics. And even one who regards a code of ethics "as unnecessary for gentlemen, and its enforcement impracticable upon those who are not," recommended, as a member of an important committee, the transfer of the following clause from the by-laws of the Association to its constitution: "Any act of special immorality or unprofessional conduct, committed by a member of this Association, shall be referred to the Committee of Arrangements, whose duty it shall be to thoroughly examine into the case, and report at the next meeting if the charges be sustained. Whereupon, by vote, the offending member may be reprimanded or expelled; a two-thirds vote being required for expulsion, a plurality being sufficient for reprimand." This is a law of the Association, and has been for years. A member is liable to expulsion even, for "unprofessional conduct," while previous to its last meeting the Association had no guide or rule to define what professional conduct is. "In those days there was no king in Israel; every man did that which was right in his own eyes."

The Association appeared to be thoroughly ready for action in the matter; and therefore, but little, if any opposition was made to the appointment of a Committee to prepare a "Code of Ethics." Of the Committee it is not necessary to speak. The Code, and not its authors, should engage our attention. It may not be amiss to state that the senior member of the Committee had read an article on Professional Ethics at the preceding annual meeting, and had carefully reconsidered the paper in regard to any modification that might seem advisable with reference to its adoption by the Association. Another member had lately acted as a member of a similar committee in the "Ohio State Dental Society," and hence, his thoughts were somewhat matured. The experience and general fitness of the other member will be universally recognized; but, unfortunately, as he stated in open society, the Committee and the Association were deprived of his valuable services in the preparation of the document. The other members, however, had the gratification of his cordially expressed approval of the report, and the appending of his signature to it. The Committee was appointed the morning of the first day, and the "Code" was adopted the evening of the fourth. It was deliberately prepared, deliberately discussed, and as deliberately adopted.

But, after all, there is no doubt that the "Code of Ethics" is very imperfect. Human documents usually are. Even the "Declaration of Independence" might be improved. The duty before us is, therefore, a plain one. Without passion, petulance, or prejudice, let defects be remedied as promptly as they are demonstrated. If important principles have been omitted, let them be added; if anything is contained that should be omitted, let it be stricken out. Let all be done cordially and frankly. Let nothing be done to gratify personal feeling; and when anything is done, let those who are fairly out-voted gracefully acquiesce.

